# A Perivascular Epithelioid Cell Tumor in the Ascending Colon: A Rare Case Involving a Patient With Tuberous Sclerosis

**DOI:** 10.7759/cureus.79061

**Published:** 2025-02-15

**Authors:** Kai Seharada, Masato Kitazawa, Satoshi Nakamura, Yuta Yamamoto, Yuji Soejima

**Affiliations:** 1 Surgery, Shinshu University School of Medicine, Matsumoto, JPN

**Keywords:** gastrointestinal tract tumors, laparoscopic colon resection, perivascular epithelioid cell tumor, submucosal tumor, tuberous sclerosis

## Abstract

Tuberous sclerosis (TSC) is a genetic disorder characterized by the development of benign tumors in various organs such as the brain, heart, lungs, kidneys, skin, and gastrointestinal tract. Perivascular epithelioid cell tumors (PEComas) are rare mesenchymal tumors associated with TSC. Primary gastrointestinal PEComas are uncommon. This report describes a PEComa in the ascending colon of a patient with TSC and presents a review of the relevant literature. A 33-year-old female patient with a history of TSC presented with a 30-mm mass in the ascending colon that had remained stable for two years. A colonoscopy revealed a 30-mm submucosal tumor in the ascending colon, and non-neoplastic biopsy results were observed. Contrast-enhanced computed tomography revealed a 30-mm mass in the ascending colon with early contrast enhancement. After considering the differential diagnoses of PEComas, gastrointestinal stromal tumors, and leiomyomas/leiomyosarcomas, laparoscopic ileocecal resection was performed. A white extramural tumor that was covered with a capsule was observed in the ascending colon intraoperatively. The histopathological analysis results suggested a complex array of spindle cells. The immunohistochemistry results for alpha-smooth muscle actin, desmin, and human melanoma black (HMB)-45 were positive, and those for c-kit and S100 were negative, thus confirming the diagnosis of a PEComa. This case highlights the importance of considering PEComas when patients with TSC present with submucosal gastrointestinal tumors and the need for careful diagnostic evaluations of such cases.

## Introduction

Perivascular epithelioid cell tumors (PEComas) comprise a group of rare mesenchymal tumors such as angiomyolipomas of the kidney and liver, clear-cell "sugar" tumors of the lungs and extrapulmonary sites, and lymphangioleiomyomatosis, which is associated with tuberous sclerosis (TSC) [1]. These tumors are characterized by cells associated with blood vessel walls and exhibit epithelioid morphology. Although PEComas can appear in various organs, their occurrence as primary tumors in the gastrointestinal tract is rare.

This report describes a unique case of a PEComa in the ascending colon associated with TSC. This case is significant because it contributes to the limited body of knowledge regarding gastrointestinal PEComas and emphasizes the need for awareness of this rare diagnosis among patients with TSC who present with gastrointestinal symptoms. This study aims to provide insights regarding the clinical, radiological, and pathological characteristics of PEComas in the gastrointestinal tract to enhance the understanding of these rare tumors.

## Case presentation

A 33-year-old woman began experiencing back pain two years before presentation for treatment. She had no known family history of TSC. Subsequently, her primary care physician ordered a contrast-enhanced CT examination, which revealed a 90-mm mass in the right lower pole of her kidney. After detailed evaluations, she was diagnosed with renal angiomyolipoma and subsequently underwent arterial embolization. Thereafter, CT of the head revealed a subependymal nodule. Based on these findings and clinical diagnostic criteria, the patient was diagnosed with TSC. Despite this case meeting the diagnostic criteria for TSC, the patient refused genetic testing.

**Figure 1:**
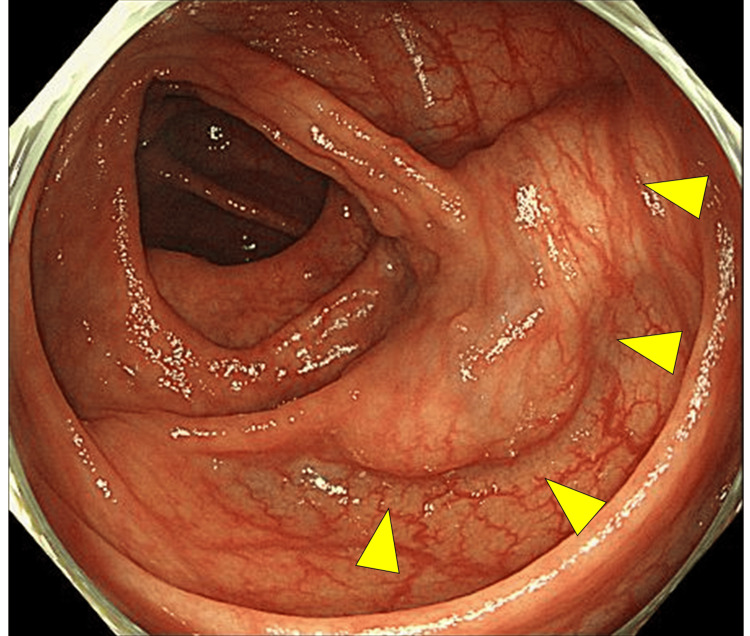
Colonoscopy A submucosal tumor-like elevation in the ascending colon (arrowheads) is observed.

An abdominal CT performed at the initial visit identified a mass in the ascending colon. A follow-up CT performed six months after arterial embolization for the renal tumor showed no significant change in size or appearance. A subsequent colonoscopy revealed an elevated submucosal tumor-like lesion in the same region. A boring biopsy was performed, but histopathological examination revealed only normal mucosa, and the sample was classified as Group 1. Given the lesion’s characteristics and size (40 mm), surgical intervention was considered a relative indication for suspected submucosal tumors of the colon. Subsequently, the patient was referred to our department for surgical management. Her medical history included uterine myoma and endometriosis, and the blood test results did not indicate elevated tumor markers. A colonoscopy confirmed the presence of a submucosal tumor-like mass in the ascending colon (Figure 1).

**Figure 2:**
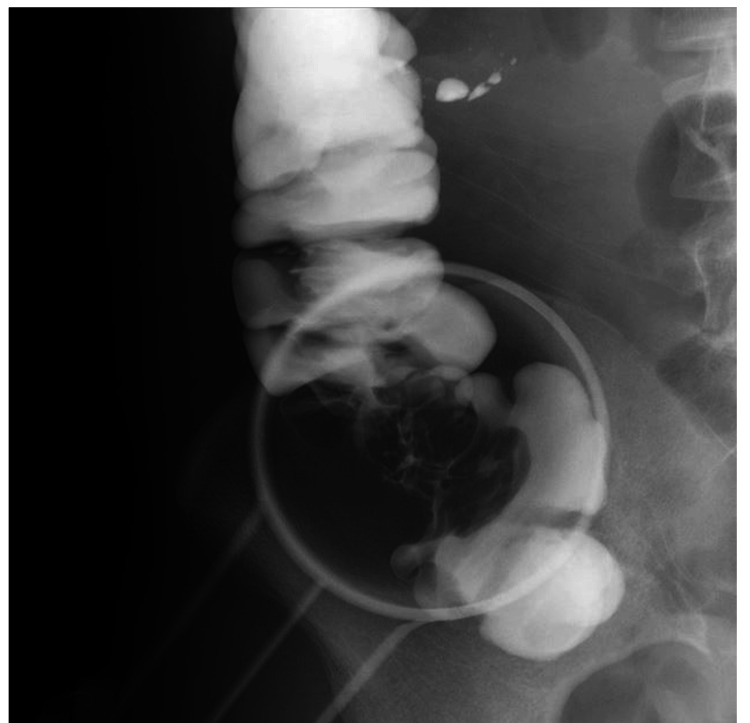
Gastrointestinal Gastrografin radiography of the ascending colon A filling defect in the ascending colon was observed on gastrointestinal Gastrografin radiography.

**Figure 3:**
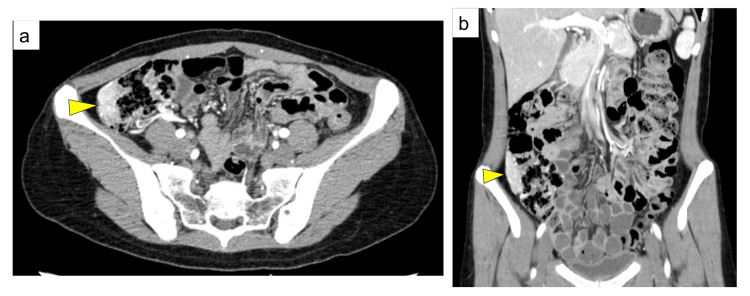
Computed tomography (a) Transverse computed tomography shows a tumor circumferentially involving the right wall of the ascending colon (arrowhead). (b) Coronal computed tomography reveals a 40-mm tumor extending along the craniocaudal axis in the ascending colon (arrowhead), with early contrast enhancement suggesting hypervascularity.

Imaging of the colon with Gastrografin revealed a shadow defect in the ascending colon opposite to the ileocecal valve (Figure 2). Contrast-enhanced abdominal CT showed a 40-mm tumor in the ascending colon with early contrast enhancement (Figure 3).

**Figure 4:**
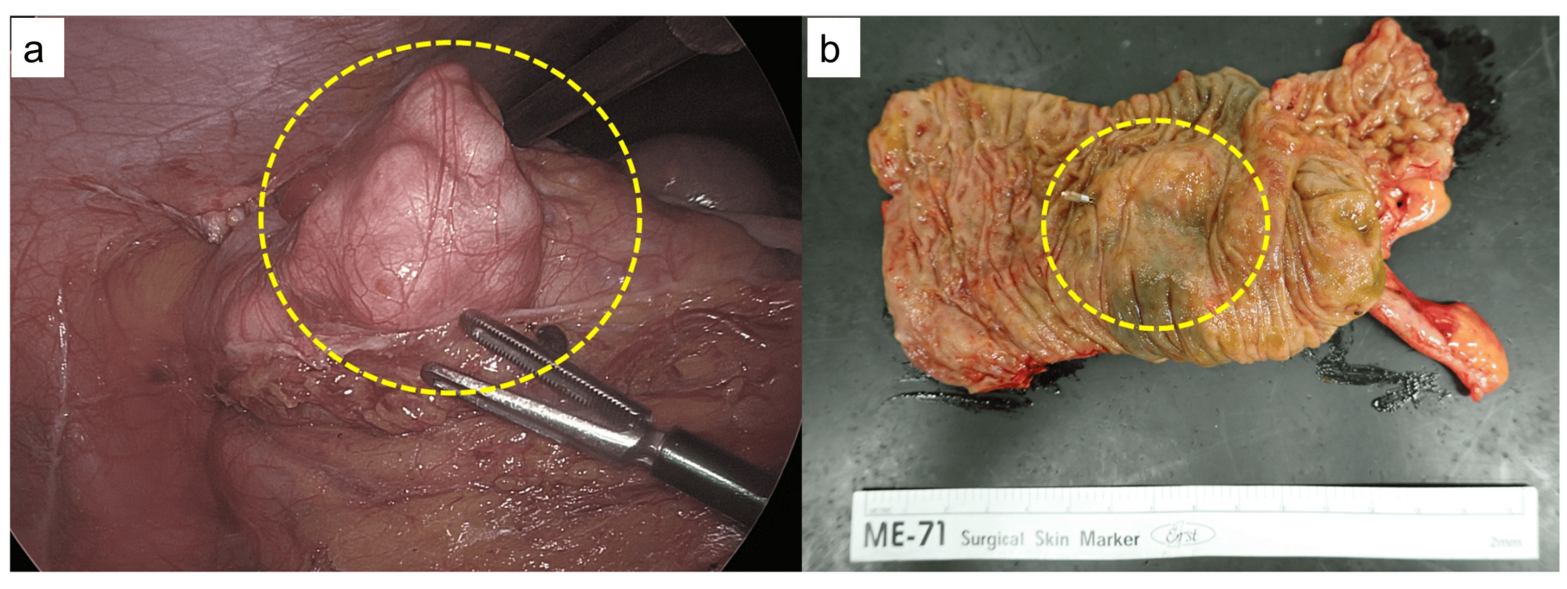
Intraoperative findings and resected specimen (a) A whitish tumor protruding extramurally was observed in the ascending colon (dashed circle). (b) In the resected specimen, a submucosal tumor-like elevation was noted in the ascending colon, while the mucosa remained intact (dashed circle).

Subsequently, laparoscopic ileocecal resection was performed six months after the first visit, and a white tumor protruding from the wall of the ascending colon was observed (Figure 4a). In the resected specimen, a submucosal tumor-like elevation was noted in the ascending colon, while the mucosa remained intact (Figure 4b).

**Figure 5:**
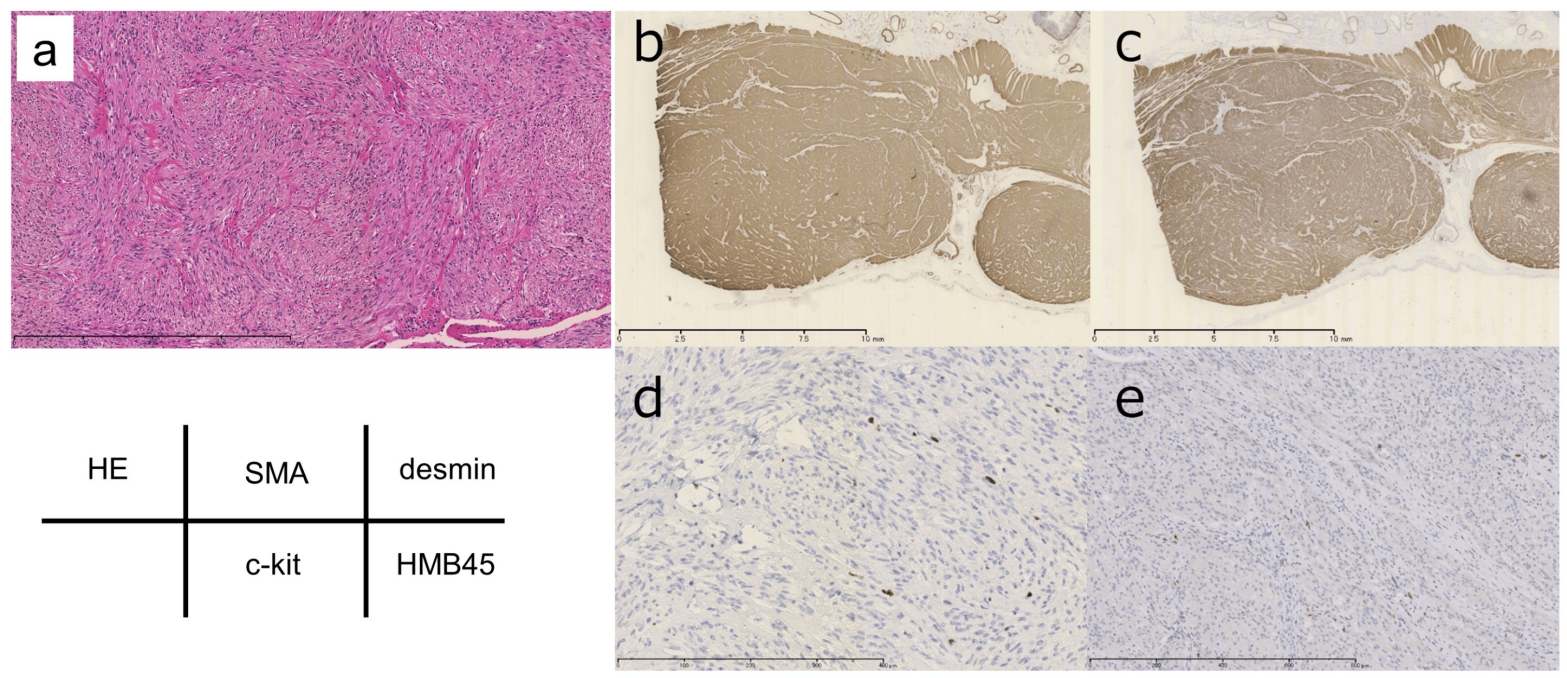
Pathological findings (a) Hematoxylin and eosin (HE) staining indicating a complex array of spindle-shaped cells, (b) positive staining for smooth muscle actin, (c) positive staining for desmin, (d) negative staining for c-kit, and (e) positive staining for human melanoma black (HMB)-45.

The histopathological examination revealed a complex network of spindle-shaped cells (Figure 5a). The immunostaining results were as follows: smooth muscle actin, positive (Figure 5b); desmin, positive (Figure 5c); c-kit, negative (Figure 5d); S100 protein, negative; and HMB-45, positive (Figure 5e). The Ki-67 labeling index was 2% to 3%, indicating a low proliferation rate, and the features were not suggestive of high-grade malignancy. Therefore, a PEComa was diagnosed. The postoperative course was uneventful, and the patient did not experience any complications. The patient was discharged on postoperative Day 7. Additionally, the patient has been followed for three years postoperatively without any recurrence or progression of the disease.

## Discussion

PEComas are rare mesenchymal tumors that occur in various organs, including the kidneys, lungs, and uterus, and originate from perivascular epithelial cells [1]. In 1996, Zamboni et al. proposed the term “PEComa” for pancreatic tumors that are closely associated with blood vessels and contain epithelial cells that exhibit smooth muscle and melanocyte differentiation [2]. Since then, PEComas have been identified at nearly all anatomical sites encompassing visceral and somatic soft tissues and, although rare, bones [3]. Patients with TSC often present with angiomyolipomas or lymphangioleiomyomatosis; however, PEComas in the gastrointestinal tract are uncommon [4]. Clinically, PEComas may manifest as abdominal pain, melena, obstruction, weight loss, and anemia; however, some cases may be asymptomatic [1]. A PEComa is a rare mesenchymal tumor with distinctive characteristics that are observed during imaging. Contrast-enhanced CT typically shows PEComas with uniform contrast enhancement outlining the mass with a well-defined border. Magnetic resonance imaging often shows these lesions as hypointense to isointense on T1-weighted images, and they have a heterogeneous hyperintense pattern on T2-weighted images. However, the features of PEComas observed with CT and magnetic resonance imaging are generally non-specific; therefore, determining a definitive diagnosis based on these imaging modalities alone is challenging. This was exemplified by our case involving a patient with TSC and a mass in the ascending colon that was incidentally discovered on a CT image despite an asymptomatic presentation. This underscores the importance of considering the clinical context and additional diagnostic methods when evaluating potential cases of PEComas.

We searched PubMed using the keywords “PEComa” and “gastrointestinal tract” and found 20 case reports involving 11 female and nine male patients with a median age of 40.5 years (range, 12-71 years) [4-23] (Table 1). The most common anatomical site of involvement was the colon (10 patients; 50%). Tumors were observed in the small intestine in four patients (20%); two cases occurred in the ileum and one occurred in the duodenum. The preoperative diagnosis is difficult and often confirmed by the postoperative pathology examination results. For this case, the pathology examination results included epithelial-like cells and spindle-shaped cells encompassing the blood vessels. Immunohistological results typically indicate positive malignant melanoma-related markers (HMB-45 and Melan-A) and muscle lineage markers. Although these tumors are generally benign, some studies have defined malignancy as a tumor diameter larger than 5 cm, an infiltrative tendency, nuclear atypia, high cell density, more than one mitotic figure per 50 high-power fields, necrosis, and vascular invasion. There is no established definitive treatment for PEComas; however, surgical resection is considered the most effective treatment approach. mTOR inhibitors (e.g., sirolimus, everolimus) and immune checkpoint inhibitors (e.g., pembrolizumab) are also considered for cases with malignant findings; however, evidence supporting their effectiveness remains limited [24-26].

**Table 1:** Review of case reports on gastrointestinal perivascular epithelioid cell tumors IHC: immunohistochemistry, HMB-45: human melanoma black 45; SMA: smooth muscle actin, NR: not recorded, NER: no evidence of recurrence

Authors	Year	Age, years	Sex	Location	Size, mm	Treatment	Metastasis		IHC					Prognosis
									HMB45	Melan-A	SMA	Desmin	S100	
Freeman HJ et al. [4]	2010	17	F	Sigmoid colon	60	Sigmoid resection	－		+	NR	－	－	－	NER at 15 years
Mitteldorf CA et al. [5]	2010	71	M	Stomach	30	Partial gastrectomy	－		－	+	+	NR	－	NER at 19 months
Unluoglu S et al. [6]	2012	36	M	Ileum	20	Abscess was drained and double ileostomy	－		+	+	+	－	－	NER at 10 months
Scheppach W et al. [7]	2013	23	M	Cecum	50	Right hemicolectomy and left hemihepatectomy	+	liver	+	NR	NR	－	－	Dead after 23 months
Kanazawa A et al. [8]	2014	55	F	Rectum	25	Trans anal resection	－		+	－	－	－	－	NER at 12 months
Chen Z et al. [9]	2015	27	M	Duodenum	82	Duodenectomy	－		+	+	NR	+	NR	NER at 4 months
Yamada S et al. [10]	2015	39	M	Stomach	73	Partial gastrectomy	－		+	+	+	+	－	NR
Iwamoto R et al. [11]	2016	42	F	Descending colon	50	Left hemicolectomy	－		+	－	NR	－	－	NER at 5 months
Acosta Materán RV et al. [12]	2016	19	M	Ileum	27	Laparotomy in which the proximal ileum	－		－	－	NR	+	NR	NER at 6 months
Shin SA et al. [13]	2016	62	F	Stomach	50	Laparoscopic wedge resection of stomach	－		+	－	+	+	－	NER at 7 years
Marano A et al. [14]	2019	55	M	Stomach	65	Robotic wedge resection	－		+	+	+	+	－	NER at 11 months
Cheng HC et al. [15]	2021	17	F	Sigmoid colon	35	Circumferential resection of the tumor	－		+	－	NR	NR	NR	NER at 24 months
Yeon HJ et al. [16]	2021	45	F	Rectum	20	Robot-assisted low anterior resection	－		+	NR	NR	NR	NR	NER at 2 years
Erginoz E et al. [17]	2021	28	F	Rectosigmoid and ileocecal	160	Proctosigmoidectomy	－		+	NR	+	+	－	NER at 1 year
Razak O A et al. [18]	2021	69	F	Cecum	NR	Laparoscopic ileocecectomy	－		+	NR	+	NR	+	NER at 48months
Kou L et al. [19]	2022	12	F	Transverse colon	50	Partial transverse colon resection	－		+	NR	－	－	－	NER at 6 months
Pereira K et al. [20]	2022	17	M	Sigmoid colon	20	Endoscopically removed	－		+	－	－	－	NR	NR
Chen Q et al. [21]	2023	55	F	Ascending colon	120	Tumor resection	－		NR	NR	NR	NR	NR	NER at 2 months
Yan H et al. [22]	2023	47	F	Sigmoid colon	20	Endoscopic mucosal resection	－		+	－	－	－	－	NER at 3 months
Sugimura N et al. [23]	2024	64	M	Transverse colon	4	Cold snare polypectomy	－		－	－	+	NR	NR	NR
Present case	2025	33	F	Ascending colon	40	Laparoscopic ileocecal resection	－		+	NR	+	+	－	NER at 12 months

## Conclusions

This case emphasizes the need to consider PEComas when patients present with TSC and gastrointestinal symptoms. An accurate diagnosis often requires both imaging and histopathological analyses because the preoperative identification of these tumors is difficult. Although surgical resection is the main treatment, chemotherapy and immunotherapy are considered for malignant cases. These valuable insights enhance the limited knowledge of gastrointestinal PEComas, thus improving their future diagnosis and management.
